# Gene representation in scRNA-seq is correlated with common motifs at the 3′ end of transcripts

**DOI:** 10.3389/fbinf.2023.1120290

**Published:** 2023-05-15

**Authors:** Xinling Li, Greg Gibson, Peng Qiu

**Affiliations:** ^1^ The Wallace H. Coulter Department of Biomedical Engineering, Georgia Institute of Technology and Emory University, Atlanta, GA, United States; ^2^ School of Biological Sciences, and Center for Integrative Genomics, Georgia Institute of Technology, Atlanta, GA, United States

**Keywords:** 10X, single-cell RNA sequencing, bulk RNA-seq, data integration, comparison, dropouts, pathway analysis, motif discovery

## Abstract

One important characteristic of single-cell RNA sequencing (scRNA-seq) data is its high sparsity, where the gene-cell count data matrix contains high proportion of zeros. The sparsity has motivated widespread discussions on dropouts and missing data, as well as imputation algorithms of scRNA-seq analysis. Here, we aim to investigate whether there exist genes that are more prone to be under-detected in scRNA-seq, and if yes, what commonalities those genes may share. From public data sources, we gathered paired bulk RNA-seq and scRNA-seq data from 53 human samples, which were generated in diverse biological contexts. We derived pseudo-bulk gene expression by averaging the scRNA-seq data across cells. Comparisons of the paired bulk and pseudo-bulk gene expression profiles revealed that there indeed exists a collection of genes that are frequently under-detected in scRNA-seq compared to bulk RNA-seq. This result was robust to randomization when unpaired bulk and pseudo-bulk gene expression profiles were compared. We performed motif search to the last 350 bp of the identified genes, and observed an enrichment of poly(T) motif. The poly(T) motif toward the tails of those genes may be able to form hairpin structures with the poly(A) tails of their mRNA transcripts, making it difficult for their mRNA transcripts to be captured during scRNA-seq library preparation, which is a mechanistic conjecture of why certain genes may be more prone to be under-detected in scRNA-seq.

## Introduction

Single-cell RNA-sequencing (scRNA-seq) allows the dissection of gene expression heterogeneity at single-cell resolution (Chen et al., 2019a), which can give insights into the existence and behavior of different cell types (Pennisi, 2018). In general, scRNA-seq technologies can be categorized into two major types: droplet-based and plate-based (Baran-Gale et al., 2018). Droplet-based scRNA-seq system includes Drop-seq (Macosko et al., 2015), inDrop (Klein et al., 2015), and 10X Chromium (Kitzman, 2016), and plated-based scRNA-seq system includes SMART-seq and SMART-seq2 (Picelli et al., 2013). Regardless of the technology, scRNA-seq data is often highly sparse. In a typical gene-cell count matrix in scRNA-seq analysis, >90% of the elements are zeros. Some of those zeros are biologically meaningful signals, such as a cell type specific marker gene showing zero expression count in cells belonging to other cell types. Meanwhile, some of those zeros represent technical issues, such as an expressed gene in a cell not being captured and hence undetected due to technical limitations. The fact that not all zeros in scRNA-seq data are problematic has been supported by multiple published studies (Kim et al., 2020; Qiu, 2020; Svensson, 2020).

Many computational methods and pipelines for scRNA-seq include components of gene selection and dimension reduction to address the high sparsity of the data. Selection of highly variable genes enables subsequent analysis to focus on genes whose zeros counts are more enriched by biologically meaningful zeros and less affected by the technical limitations (Qiu, 2020). In dimension reduction techniques [e.g., PCA (Friedman et al., 2001), t-SNE (Kobak and Berens, 2019) and UMAP (Becht et al., 2018)], the reduced dimensions are derived by linear or non-linear combinations of genes, which borrow strength across genes to reduce the sparsity. Methods that adopted these approaches include Seurat (Butler et al., 2018), TSCAN (Ji and Ji, 2016), and STREAM (Chen et al., 2019b). In addition, many imputation algorithms have been developed to generate improved versions of the data with lower sparsity, such as scImpute (Li and Li, 2018), MAGIC (van Dijk et al., 2018), RESCUE (Tracy et al., 2019), and SAVER (Huang et al., 2018). Many of these imputation tools also adopt gene selection and dimension reduction, so that they can robustly identify gene-gene similarities or cell-cell similarities and use these relationships to impute the data (Hou et al., 2020). Furthermore, an opposite view of the sparsity has been presented in two algorithms, co-occurrence clustering (Qiu, 2020) and HIPPO (Kim et al., 2020), which demonstrated that the sparsity pattern of scRNA-seq data can be an extremely useful signal to accurately identify cell clusters and cell types. Therefore, the literature and research community has not formed a consensus of best practice to handle the sparsity of scRNA-seq data.

In the literature, the high sparsity in scRNA-seq is often referred to as dropout. The term dropout was introduced to describe technical failures that may cause a highly expressed gene to be undetected (Kharchenko et al., 2014) However, in widespread discussions, the use of this terminology has been inconsistent. Dropout sometimes refers to zeros caused by technical issues so that expressed genes are undetected, sometimes refers to all observed zeros in the data, and sometimes refers to the fact that not all mRNA molecules in the biological sample are captured which causes all genes to be under-detected to some extent (Sarkar and Stephens, 2021). In this paper, our usage of the term dropout aligns with the third meaning above, and we are interested in examining whether there exist genes that are more prone to be under-detected in scRNA-seq.

In order to develop methods to address dropouts or under-detection in scRNA-seq, it is important to understand the factors that contribute to the dropouts. Recent studies have suggested that 3′-UTR length, compartment, transcript count, and differential expression levels (Andrews and Hemberg, 2019; Lipnitskaya et al., 2022) may play roles in the dropouts of scRNA-seq. For example, genes with shorter 3′-UTR length have larger quantitative difference between gene expression in matched scRNA-seq and bulk RNA-seq experiments (Lipnitskaya et al., 2022). In addition, choice of technology platform can also affect dropouts. For example, comparisons between SMART-seq2 and 10X Chromium showed that 10X Chromium had more noise and a higher dropout rate (Wang et al., 2021). However, these previous studies involved relatively small numbers of samples, which led to conclusions with limited scope and generality. In this study, we collected paired bulk RNA-seq and scRNA-seq samples from diverse data sources and diverse biological contexts, and used this data to investigate whether there exist genes that are more prone to be under-detected in scRNA-seq, and if yes, what commonalities those genes may share.

## Results

### Paired bulk RNA-seq and scRNA-seq data

Through extensive literature search, we have identified eight datasets with paired bulk RNA-seq data and scRNA-seq data available for the same samples. A summary of these datasets is listed in the Materials and Methods. In total, we have paired bulk RNA-seq data and scRNA-seq data for 53 samples. The samples originated from diverse biological contexts, including fibroblasts, trachea, women reproductive system, breast cancer, and cancer cell lines.

For each GEO bulk RNA-seq dataset, median-of-ratios normalization was performed followed by log transformation. The scRNA-seq data for each sample was preprocessed separately, with library size normalization followed by log transformation. Then, a pseudo-bulk RNA-seq profile was calculated for each sample, by averaging the scRNA-seq expression data across all cells in the sample. Next, for each sample pair, the normalized bulk RNA-seq and single-cell based pseudo-bulk expression of overlapping genes among the two data types were identified. With these preprocessing steps, for each of the 53 samples, we obtained one bulk RNA-seq profile and one pseudo-bulk RNA-seq profile for the overlapping genes. Then quantile normalization was performed for the bulk RNA-seq profiles.

**FIGURE 1:**
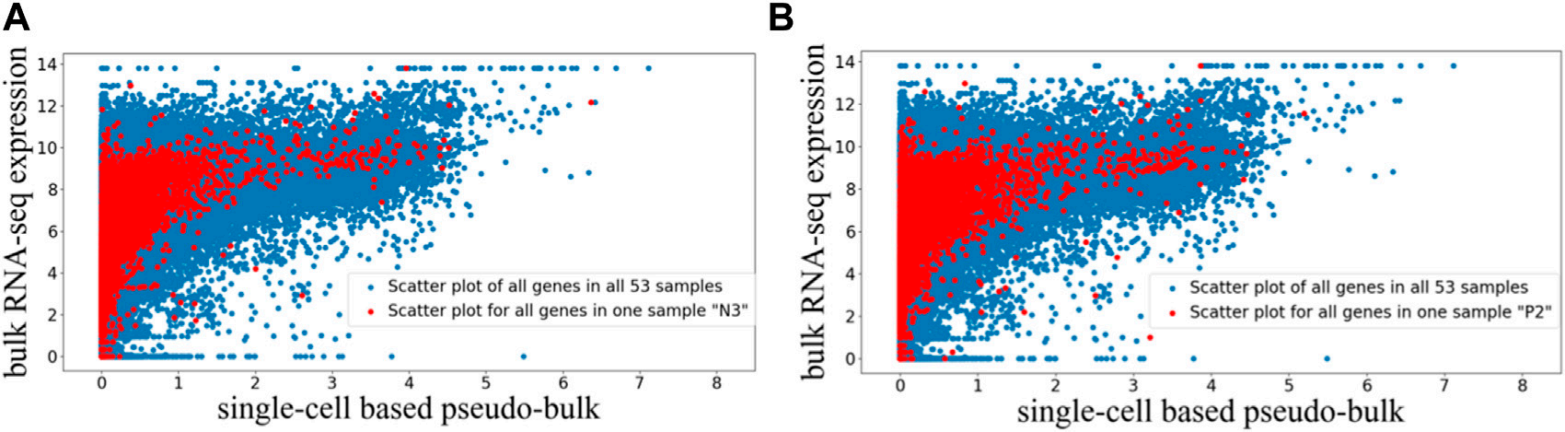
Scatter plot visualization of paired bulk and pseudo-bulk data for 53 samples. Each dot is expression of one gene in one sample, so there are 36,362 genes *53 dots in one scatter plot. **(A)** Scatter plot of all genes in all 53 samples in blue, overlaid with scatter plot for all genes in one sample “N3” in red. **(B)** Scatter plot of all genes in all 53 samples in blue, overlaid with all genes in another sample “P2” in red.

The preprocessed paired bulk and pseudo-bulk data were visualized using the scatter plots in Figure 1, where each dot represents expression data of one gene in one sample. In Figure 1A, we visualized the paired bulk and pseudo-bulk data for all 53 samples (blue), and overlaid with the paired bulk and pseudo-bulk data for one of the samples (red). In Figure 1B, the same visualization was used to highlight another sample in the context of all samples. Visualizations highlighting other samples (not shown) looked similar to Figures 1A, B. Based on these scatter plots, we can see the general correlation between bulk RNA-seq and scRNA-seq data, which is expected. To justify the choice of quantile normalization for processing the bulk expression data, we tried to alter our analysis pipeline by removing quantile normalization for bulk RNA-seq data, and we noticed that the alignment of normalized bulk data across samples was poor. For example, as shown in Supplementary Figure S1, without quantile normalization, the range of normalized bulk RNA-seq data for the two highlighted samples showed marked difference. Therefore, quantile normalized is needed for the bulk RNA-seq data. Across the 53 paired samples, the Pearson correlation between the two data types has mean and standard deviation of 0.385 ± 0.063, while the Spearman correlation has mean and standard deviation of 0.849 ± 0.049. The Pearson correlation is lower than the Spearman correlation, which is expected, because the relationship between the two data types is not linear as shown in Figure 1. In addition, we can see that the preprocessing steps were able to properly align the 53 bulk RNA-seq expression profiles across different datasets, and also properly align the 53 pseudo-bulk expression profiles, so that we can compare across these samples to identify genes that tend to be under-detected in scRNA-seq relative to bulk RNA-seq.

### Genes that are consistently under-detected in scRNA-seq

**FIGURE 2:**
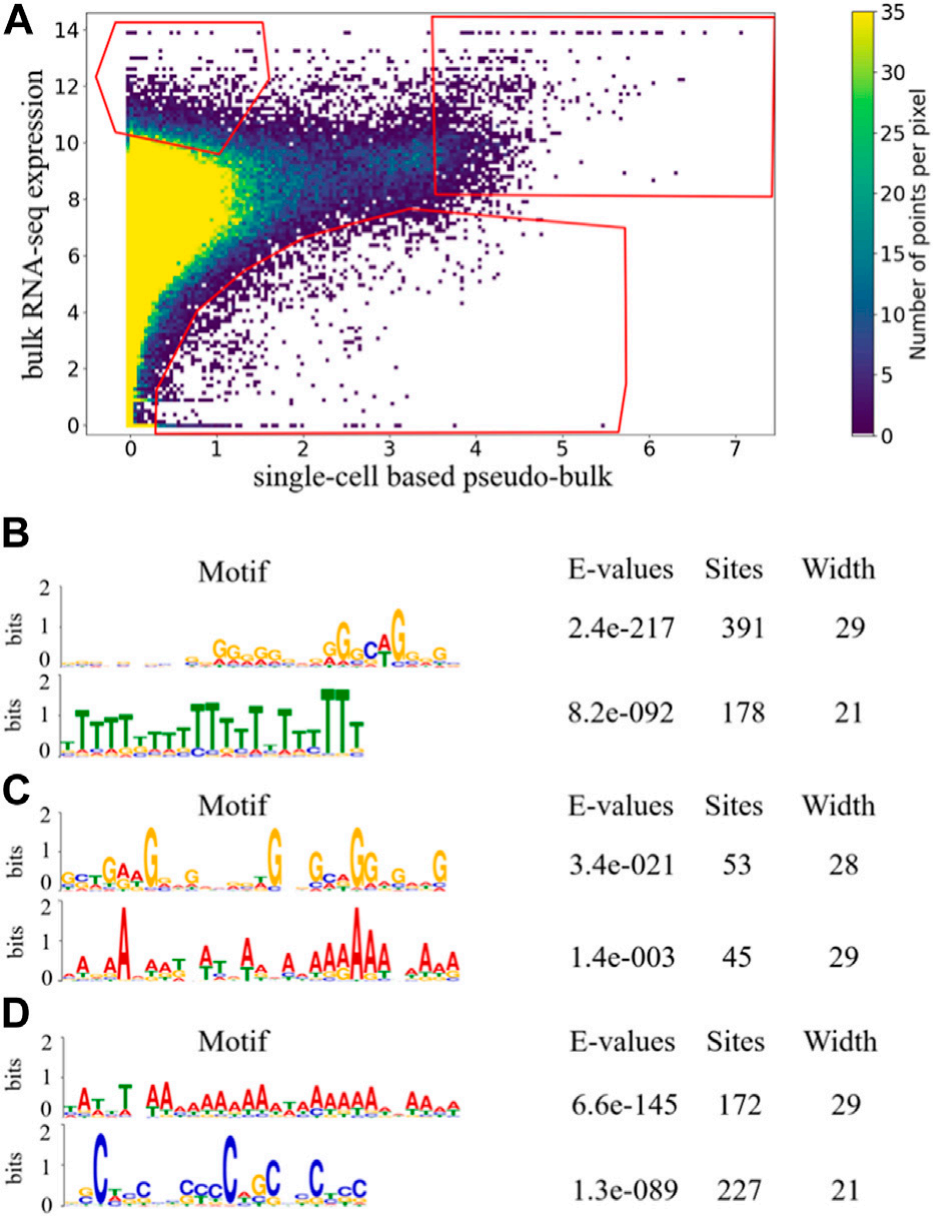
Density plot of scatterplot of 53 paired samples with gates indicating candidate genes in three aspects. The gates were selected based on data distribution of 53 paired sample such that the gate in the upper-left corner represents the genes that are under-detected in scRNA-seq experiments compared to bulk RNA-seq experiments, the gate in the upper-right corner represents the genes that are highly expressed in both bulk RNA-seq and scRNA-seq experiments, and the gate in the bottom corner represents the genes that are over-detected in scRNA-seq experiments compared to bulk RNA-seq experiments **(A)**. Significantly enriched motifs in last 350bp of the longest transcripts of genes that occurred more than once in each gate are shown for upper-left gate **(B)**, upper-right gate **(C)**, and bottom gate **(D)**.

In order to identify genes that are more prone to dropout or under-detection in scRNA-seq, we examined whether there exist genes that repeatedly appeared in the upper-left corner of the scatter plot in Figure 1, which compared bulk RNA-seq expression profiles and pseudo-bulk expression profiles derived from scRNA-seq. We visualized the scatter plot as a density plot in Figure 2A, and manually drew a gate (region-of-interest) in its upper-left corner. We positioned the gate to avoid high density regions, so that genes falling into the gate represented outlier cases where expressions detected by scRNA-seq were much lower than expressions detected by bulk RNA-seq. If a gene appeared in the gate multiple times, this gene was consistently under-detected in scRNA-seq experiments compared to bulk RNA-seq experiments for multiple of the 53 samples.

The top 15 genes that most frequently appeared in the upper-left gate are listed in Table 1, along with their numbers of appearances which ranged from 26 to 44. This suggests that out of the 53 samples with paired bulk and single-cell data, these genes were frequently under-detected by scRNA-seq in more than half of the samples. Therefore, indeed, there seem to exist genes that are consistently under-detected in scRNA-seq experiments for many samples. The 15 genes include AHNAK, EIF4G2, XIST, CSDE1, DST, DDX17, FN1, SRRM2, FLNA, YWHAZ, COL3A1, ITGB1, PRRC2C, COL1A1, and GNAS. AHNAK encodes a protein involved in diverse processes such as blood-brain barrier formation, cell structure and migration, cardiac calcium channel regulation, and tumor metastasis (Stelzer et al., 2011). DDX17 encodes a DEAD box protein. DEAD box proteins are implicated in a number of cellular processes involving alteration of RNA secondary structure, such as translation initiation, nuclear and mitochondrial splicing, and ribosome and spliceosome assembly (Stelzer et al., 2011). EIF4G2 functions as a general repressor of translation by forming translationally inactive complexes (Stelzer et al., 2011).

**TABLE 1:** List of top 15 genes that are most frequently under-detected in scRNA-seq and their frequencies.

Gene name	Frequency of occurrence among 53 sample pairs
AHNAK	44
EIF4G2	43
XIST	39
CSDE1	35
DST	35
DDX17	34
FN1	34
SRRM2	31
FLNA	30
YWHAZ	28
COL3A1	27
ITGB1	27
PRRC2C	27
COL1A1	26
GNAS	26

Given the diversity of biological contexts of the 53 samples and the possibility that any given gene may only be expressed in a subset of those contexts, we broadened our criterion for under-detected genes in scRNA-seq and considered all genes that occurred more than once in the upper-left gate in Figure 2A. The total number of dots in the upper-left gate is 3,363, with each dot representing one gene in one sample. There were 468 unique genes that appeared more than once in the upper-left gate, which is roughly 3 times more than expected according to the hypergeometric test. The fact that hundreds of genes appeared more than once in the upper-left gate is interesting. Gene set enrichment analysis showed that those genes were involved in multiple KEGG pathways related to cancer. For example, several significantly enriched KEGG pathways include proteoglycans in cancer (FDR = 1.24E-13), pathways in cancer (FDR = 0.02), and microRNAs in cancer (FDR = 0.036). This is expected because 29 out of the 53 sample were generated from cancer patients or cancer cell lines.

To examine sequence-based commonalities among the genes that appeared more than once in the upper-left gate in Figure 2A, we searched for enriched motifs in the last 350 bp of those genes using the MEME Suite (Bailey et al., 2009), and observed two motifs that were significantly enriched with small E-value and large number of sites (Figure. 2B). For the enriched poly(T) motif, the position-weight visualization showed that the bit score for most of the positions were high. For many positions, the bit score was over 50%, and for some of the positions the score reached 80%. This indicated that there was relatively high certainty about the enrichment of T at most of the positions within the motif, forming a consecutive block of T’s. In contrast, for the enriched poly(G) motif, the bit score for most of the positions were low, indicating that the certainty of having G at the positions was low. In addition, the G’s do not form long consecutive blocks. Therefore, even though the number of sites of the poly(G) motif was large and its E-value was significant, the poly(G) motif was not as strong as the poly(T) motif. Since there is no obvious mechanism associated to the poly(G) enrichment, we conjectured that the poly(T) motif toward the tails of genes under-detected in scRNA-seq may be able to form hairpin structures with the poly(A) tails of their mRNA transcripts, making it difficult for their mRNA transcripts to be captured during the capturing step of scRNA-seq library preparation, which is a mechanistic conjecture of why those genes may be more prone to be under-detected in scRNA-seq.

### Genes consistently highly expressed in both bulk RNA-seq and scRNA-seq

As a comparison, we manually drew another gate in the upper-right corner of the density plot in Figure 2A, and examined whether there exist genes that were consistently highly expressed in both bulk RNA-seq and scRNA-seq. The manually drawn gate was positioned such that the number of genes within the gate was comparable to the number of genes within the gate in the upper-left corner. In addition, we positioned the gate to avoid high density regions, so that genes falling into the gate represented outlier cases in Figure 2A where expression detected in both bulk RNA-seq and scRNA-seq are high. If a gene appeared in the upper-right gate multiple times, this gene consistently showed high expression in both bulk RNA-seq and scRNA-seq for multiple of the 53 samples. The total number of dots in the upper-right gate is 1,451, among which 96 unique genes appeared more than once.

For the top 15 genes with highest frequency of occurrences in the upper-right gate, their numbers of occurrences ranged from 29 to 50, which were more than half of the 53 samples. Given the diverse biological contexts of the 53 samples under consideration, such consistency of highly expressed genes was interesting. Meanwhile, since many housekeeping genes are known to be involved in diverse fundamental biological processes, such consistency of highly expressed genes was also expected. The top 15 genes with highest frequency in the upper-right gate included MALAT1, RPLP1, EEF1A1, RPL10, RPL13, RPS18, FTH1, B2M, TMSB4X, RPS4X, RPL13A, RPL32, RPS12, RPS27A, and RPL11. B2M encodes a protein which is associated with MHC class I heavy chain on the surface of nearly all nucleated cells (Stelzer et al., 2011). TMSB4X encodes a protein which is involved in cell proliferation, migration, and differentiation and it is a major cellular constituent in many tissues (Stelzer et al., 2011). EEF1A1 is expressed in brain, placenta, lung, liver, kidney, and pancreas, and it is responsible for the enzymatic delivery of aminoacyl tRNAs to the ribosome (Stelzer et al., 2011). In addition to the top 15 most frequently appeared genes, we also considered genes that appeared more than once in the upper-right gate in Figure 2A. Gene set enrichment analysis showed that those genes were involved in multiple GO terms including ribosome (FDR = 3.89E-69), cytosol (FDR = 2.55E-25), RNA binding (FDR = 1.53E-44), and protein binding (FDR = 4.01E-4), which supported our intuition that the upper-right gate is enriched for housekeeping genes required for diverse fundamental cellular processes.

Using the MEME Suite, we searched for enriched motifs among the 96 genes that appeared more than once in the upper-right gate in Figure 2A, and observed two enriched motifs with moderate number of sites, a poly(A) motif and a poly(G) motif (Figure. 2C). For both of these enriched motifs in Figure 2C, the bit scores were relatively low and did not form long consecutive blocks, suggesting that they were not as strong as the poly(T) motif enriched in the upper-left gate. It was encouraging to see that the poly(T) motif enriched in the upper-left gate was not observed in the upper-right gate, which strengthened our mechanistic conjecture of the poly(T) motif and hairpin structures may play a role in under-detection of gene expression in scRNA-seq experiments.

### Genes that appear to be over-detected in scRNA-seq

For completeness, we also attempted to identify genes that are frequently over-detected in scRNA-seq compared to bulk RNA-seq. We manually drew a third gate in Figure 2A, to define the outlier cases in the bottom-right region with low density. The gate was positioned such that the numbers of genes within each of the three gates were comparable. If a gene appeared in the bottom gate multiple times, this gene is consistently over-detected in scRNA-seq compared to bulk RNA-seq. The total number of dots in the bottom gate is 1,079, which contained 174 unique genes that appeared more than once. Comparing to the total number of dots and number of unique genes in the upper-left and upper-right gates, the average occurrence of unique genes in the bottom gate was much smaller than genes in the other two gates, indicating that much fewer genes were consistently over-detected in scRNA-seq.

For genes that appeared more than once in the bottom gate in Figure 2A, we performed motif search using the MEME Suite, and observed two highly enriched motifs (Figure. 2D). For the enriched poly(A) motif, the bit scores for various positions were moderate. For enriched poly(C) motif, the bit scores were relatively low at most of the positions, and the C’s do not form a long consecutive block. Therefore, neither of the enriched motifs in the bottom gate was as strong as the poly(T) motif enriched in the upper-left gate. Therefore, among genes that tended to be over-detected in scRNA-seq, the absence of the poly(T) motif further strengthened our conjecture that sequence-based feature may be predictive of capturing efficiency during scRNA-seq library preparation.

### Robustness of enriched sequence motifs to choices of normalization procedure

To demonstrate the robustness of the enriched sequence motifs for the genes that are under-detected in scRNA-seq, we repeated the analysis of the 53 paired samples with four choices of scRNA-seq normalization algorithms, including DESeq2 (Love et al., 2014), SCTransform (Hafemeister and Satija, 2019), Linnorm (Yip et al., 2017), and scran (Lun et al., 2016). For each choice of normalization algorithm, we generated pseudo-bulk data based on the normalized scRNA-seq data, and compared with the normalized bulk RNA-seq data using the same analysis as in Figure 2. Results of these four analyses based on different scRNA-seq normalization algorithms are shown in Supplementary Figures S2–S5. In these supplementary figures, we consistently observed that poly(T) motif was significantly enriched in upper-left gate of genes under-detected in scRNA-seq, and poly(A) motif was enriched in upper-right and bottom gates. These results suggested that our observation of motif enrichment is robust to the choice of the normalization procedure.

### Randomly paired bulk RNA-seq and scRNA-seq expression profiles

To examine the robustness of our comparison between paired bulk RNA-seq and scRNA-seq expression data, we randomly shuffled the gene expression profiles to create 53 random pairs, where each pair of bulk RNA-seq profile and pseudo-bulk profile from scRNA-seq were generated from different biological samples. With the randomly paired data, we performed the same analysis as above, and examined whether the randomly paired data would produce similar results.

**FIGURE 3:**
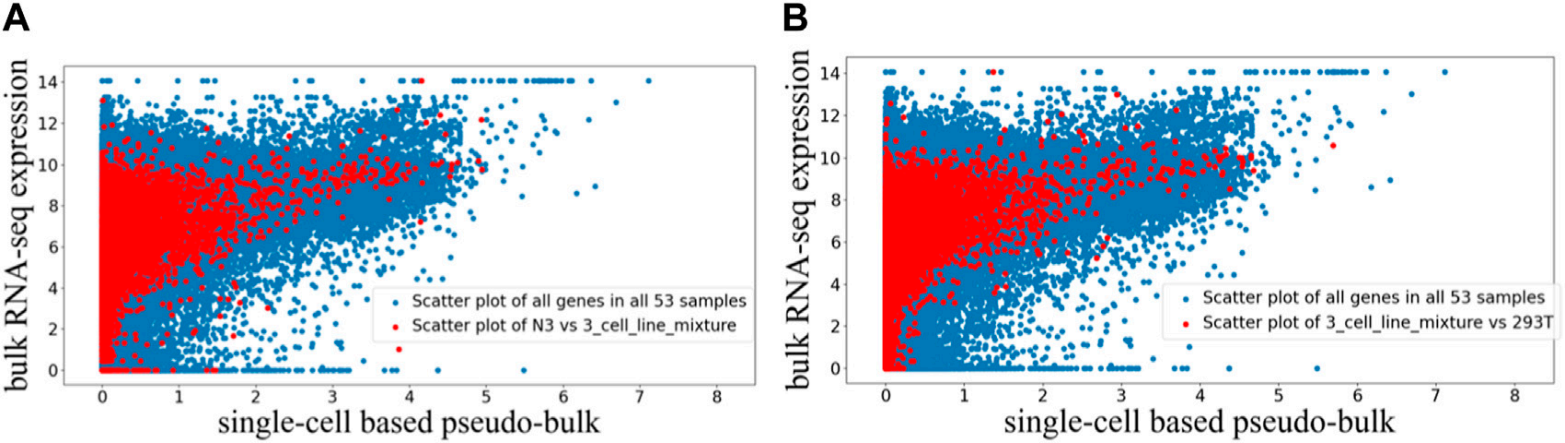
Scatter plot visualization of randomly paired bulk and pseudo-bulk data for 53 samples. Each dot is expression of one gene in one sample, so there are 37,387 genes *53 dots in one scatter plot. **(A)** Scatter plot of all 53 samples in blue, overlaid with scatter plot for one sample pair “N3 (bulk RNA-seq) vs. 3_cell_line_mixture (scRNA-seq)” in red. **(B)** Scatter plot of all 53 samples in blue, overlaid with another sample pair “3_cell_line_mixure (bulk RNA-seq) vs. 293T (scRNA-seq)” in red.

The randomly paired bulk and pseudo-bulk data were visualized using the scatter plots where each dot represents expression data of one gene in one randomly paired expression profiles (Figure 3). In Figure 3, we visualized all 53 randomly paired bulk and pseudo-bulk data (blue), and overlaid with one such random pair (red) in Figure 3A and another random pair in Figure 3B. Visualizations highlighting other random pairs (not shown) were similar to Figures 3A, 3B. Based on these scatter plots, we observed that the general correlation between bulk RNA-seq and scRNA-seq data was robust to random pairing of the data. Across the 53 randomly paired samples, the Pearson correlation between the two data types has mean and standard deviation of 0.350 ± 0.055, while the Spearman correlation has mean and standard deviation of 0.786 ± 0.058. The average correlation values were slightly lower for the randomly paired data compared to the average correlation values for the paired data.

**FIGURE 4:**
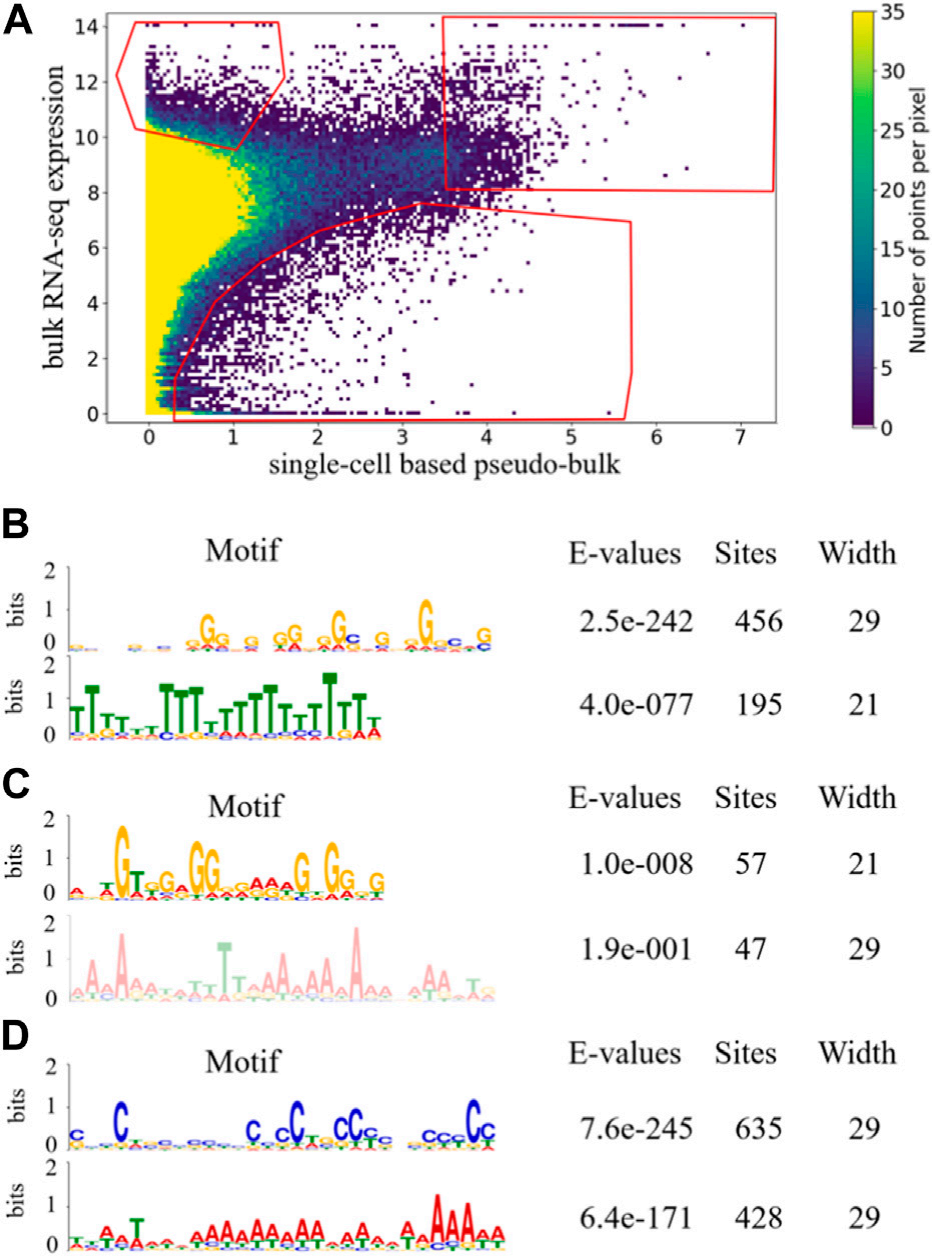
Density plot of scatter plot of 53 randomly paired samples with gates indicating candidate genes in the three aspects for one of the 100 iterations. The coordinates of gates were the same as those of the scatter plot of 53 paired sample **(A)**. Enriched motifs of last 350 bp of the longest transcripts of genes that occurred more than once in each gate are shown for upper-left gate **(B)**, upper-right gate **(C)**, and bottom gate **(D)**.

Similar to the analysis above, we manually drew three gates in Figure 4A to capture genes that tended to be under-detected in scRNA-seq, over-detected in scRNA-seq, or highly expressed in both bulk RNA-seq and scRNA-seq. The numbers of unique genes appeared more than once in the upper-left, upper-right and bottom gates in Figure 4A were 473, 97, and 507, respectively. Before random pairing, the numbers of genes appearing more than once in those three gates in Figure 2A were 468, 96 and 174. The comparable numbers for the upper-left and upper-right gates were encouraging, showing that the pattern of under-detection in scRNA-seq and the pattern of high expression in both technologies were robust to random pairing of the data. Interestingly, the number of genes appearing more than once in the bottom gate became much larger after random pairing. This was likely because the random pairing increased the variations in the scatter plot, so that more dots/genes fell into the bottom gate. This observation indicated that the pattern of over-detection in scRNA-seq was not as robust as the other two patterns of under-detections in scRNA-seq and high expression in both technologies. For each of the three gates in Figure 4A, we performed motif search to the last 350 bp of genes that appeared more than once. Similar to the results before random pairing, for genes that occurred in the upper-left gate in Figure 4A, poly(T) motif was significantly enriched while poly(A) motif was not observed. In contrast, for genes that appeared more than once in the other two gates in Figure 4A, poly(T) motif was not enriched. It was encouraging to see that the motif enrichment that led to our mechanistic conjecture on detection in scRNA-seq was robust to random pairing of the data.

### Comparison of frequently appearing genes in paired and randomly paired data

As a further comparison between the paired and randomly paired RNA-seq and scRNA-seq data, we examined genes that frequently appeared in the three gates, ≥20 times out of the 53 samples under consideration. For each gate in Figure 2A based on the paired data, we computed the ratio between the number of genes that appeared ≥20 times and the total number of unique genes, and listed the ratios in the first row of Table 2. We also calculated these ratios for the gates in Figure 4A based on the randomly paired data, as shown in the second row of Table 2. In addition, we performed 100 iterations of the random pairing, which allowed us to quantify the variation of these ratios in the second row of Table 2 when the bulk RNA-seq and scRNA-seq data were randomly paired. Once again, in terms of number of genes frequently appearing in the three gates, we observed that the results were robust with respect to random pairing of the data.

**TABLE 2:** Percentage of genes that frequently appeared (≥20 times) in the three gates in the analysis of paired data, and the percentage of frequently appearing genes in the three gates in the analysis of randomly paired data.

Percentage of unique genes that occurred≥20 times	Upper-left gate	Upper-right gate	Bottom gate
Paired data	4.3%	23.2%	0.8%
Randomly paired data (100 iterations)	5.0% (±0.7%)	21.5% (±2.7%)	0.6% (±0.3%)

The percentage for frequently appearing genes in the upper-right gate was above 20%, indicating great consistency of highly expressed genes across the 53 samples from diverse biological context, which agreed with our observation that the upper-right gate was enriched for housekeeping genes required for diverse fundamental cellular processes. The percentage for frequently appearing genes in the upper-left gate was around 4%–5%, which was lower than the upper-right gate but much higher than the bottom gate, indicating that the pattern of under-detection in scRNA-seq is more consistent than over-detection in scRNA-seq.

In addition to comparing the number or percentage of frequently appearing genes, we also examined whether those frequently appearing genes were the same between paired and randomly paired data. For each gate, we computed the average intersection-over-union ratio between the sets of frequently appearing genes in the paired and randomly paired analyses, averaging across the 100 iterations of random pairing. The average intersection-over-union ratios were 0.75 and 0.66 for the upper-left and upper-right gate, indicating high overlap for those two gates in the paired and randomly paired analyses. In contrast, the average intersection-over-union ratio for the bottom gate was only 0.17, showing that frequently appearing genes in the bottom gate were quite different between the paired and randomly paired analyses, which further indicated that the pattern of over-detection in scRNA-seq is weak.

## Discussion

In this study, we analyzed paired bulk RNA-seq and scRNA-seq data from 53 samples from various biological contexts. Comparison between bulk RNA-seq and scRNA-seq data revealed genes that were consistently under-detected in scRNA-seq, and this result was robust to random pairing of the data. In addition, we observed that the frequently under-detected genes in scRNA-seq were significantly enriched by the poly(T) motif. In contrast, enrichment Tof poly(T) motif was not observed in genes consistently highly expressed in both technologies or genes that appeared to be over-detected in scRNA-seq. The motif-based observation led to our hypothesis that the poly(T) motif in genes may be able to form hairpin structures with the poly(A) tails of their mRNA transcripts, making it difficult for their mRNA transcripts to be captured during the capturing step of scRNA-seq library preparation, which is a mechanistic conjecture of why those genes may be more prone to be under-detected in scRNA-seq compared to bulk RNA-seq.

The datasets analyzed in the study not only reflected a variety of biological contexts, but also contained technical variations in experimental and computational analyses. These technical variations include choices of alignment tools, choices of reference genome, library preparations and other experimental factors. All these factors could impact the data and subsequent analyes, including the results presented in our study. An ideal situation for our study is that all samples were processed using the same experimental protocol, the same reference genome, and the same alignment software with identical version. However, this is infeasible because almost all previous datasets involved some unique details in their experimental protocols. In addition, since raw FASTQ files were unavailable for many of the bulk RNA-seq and scRNA-seq samples in this study, we were unable to obtain the raw reads to run a standardized pre-processing pipeline to derived the gene expression data for all the samples. One experimental factor in scRNA-seq, the choice between 3′ vs. 5′ library preparation protocol, presents an interesting discussion, because our motif observations and mechanistic conjecture are both relevant to the 3′ end. Among the 8 datasets included in this study, 7 were generated using 3′ protocol. The remaining dataset contained a mix of 3′ and 5′ scRNA-seq data, but did not provide information on which samples were profiled by which protocols. Therefore, we did not distinguish 3′ vs. 5’ in our analysis. Since the above-mentioned factors were ignored in our analysis, we were effectively embracing the variations caused by those factors. Even with such variations in the data, we still observed a robust motif for the upper-left gate of genes under-detected in scRNA-seq. Therefore, these technical variations strengthened the robustness of our results.

Although the poly(T) motif was significantly enriched among genes that were frequently under-detected in scRNA-seq, there were consistently under-detected genes that lacked this motif. For example, among the top 15 genes that were most frequently under-detected in scRNA-seq as shown in Table 1, the poly(T) motif was not present in CSDE1, FLNA, FN1, DST, EIF4G2, and YWHAZ, while the remaining 9 genes contained the poly(T) motif. The mechanism of why these 6 genes were consistently under-detected in scRNA-seq is still unclear and needs further investigation.

For the genes that are repeatedly under-detected in scRNA-seq, they are less likely to be considered as highly variable genes, and thus, are less likely to drive clustering or trajectory analysis results in downstream analyses. However, recognizing such genes is important. If the goal of a research project is to investigate a specific gene which happens to be more prone to be under-detected in scRNA-seq, scRNA-seq may be a less reliable experimental strategy compared to bulk RNA-seq. When developing imputation analysis of scRNA-seq data, more attention should be paid to genes with enriched poly(T) motif. As another future direction, the bulk vs. single-cell comparison in this study can be extended to other genomic data types, such as paired ATAC-seq and scATAC-seq data for a common set of samples.

## Materials and methods

### Summary of datasets

Expression data for 53 paired bulk RNA-seq and scRNA-seq samples were obtained from 8 published GEO datasets. The paired samples were from either the same individuals, the same cell lines, or the same tissue sources. The scRNA-seq for majority of the 53 samples were generated using 10X Chromium single-cell 3’ v2 or v3 protocol. Some of the samples in one GEO dataset (GSE176078) were processed using 10X Chromium single-cell 5’ protocol, but the identity of those samples was not available. A summary of the 8 GEO datasets and their references is available in Table 3. More details about accession of individual samples and how bulk RNA-seq and scRNA-seq samples were paired for each dataset can be found in Supplementary Table S1.

**TABLE 3:** Summary of datasets.

Bulk RNA-seq and scRNA-seq datasets	Source of samples	Number of samples
GSE151202 (Li et al., 2021)	human vaginal wall from women with severe anterior vaginal prolapse	15
GSE161529 (Pal et al., 2021) and GSE161892 (Pal et al., 2021)	Breast cancer	3
GSE176078 (Wu et al., 2021)	Breast cancer	24
GSE149694 (Liu et al., 2020) and GSE150311 (Liu et al., 2020)	human fibroblasts	4
GSE108382 (Ho et al., 2018) and GSE108394 (Ho et al., 2018)	Melanoma cell line	2
GSE136148 (Dong et al., 2021)	A mixture of MDA-MB-438, MCF7, and human dermal fibroblast cell lines	1
GSE143705 (Carraro et al., 2020) and GSE143706 (Carraro et al., 2020)	Human trachea	2
GSE129240 (Zaitsev et al., 2019) and 10X website	Jurkat and 293T cell lines	2

### Data preprocessing of bulk RNA-seq

For each GEO bulk RNA-seq dataset, median-of-ratios normalization was performed by DESeq2 which accounts for factors including sequencing depth and RNA composition. Next, log transformation was performed on the normalized data. Then the overlapping genes among bulk RNA-seq and scRNA-seq of each paired sample were identified for the 53 sample pairs from the GEO datasets, and a matrix representing the normalized bulk RNA-seq expression of all overlapping genes of the 53 paired samples was created. Finally, quantile normalization was performed on this matrix.

### Data preprocessing of scRNA-seq

For scRNA-seq samples, library size normalization was performed by Seurat followed by natural log transformation and calculation of average expression of each gene across all the cells to get pseudo-bulk RNA-seq data. Then, a matrix representing the single-cell based pseudo-bulk expression of the 53 samples was created.

### Correlation analysis of bulk RNA-seq and scRNA-seq

Pearson correlation and Spearman correlation were used to calculate the relationship between normalized bulk RNA-seq and pseudo-bulk RNA-seq expression profiles of the paired and randomly paired data.

### Motif enrichment analysis

MEME Suite was used to find significant enriched sequence motifs of last 350bp of cDNA sequences of longest transcripts of candidate genes. For motif site distribution, any number of occurrences was selected for the analysis. MEME Suite reports E-value which serves as an indicator of the statistical significance of a motif. A motif with an E-value smaller than 0.05 is considered to be significant.

### Pathway analysis

DAVID (Database for Annotation, Visualization, and Integrated Discovery) was used to identify the enriched KEGG pathways, and biological process, cellular component, and molecular function GO terms. FDR value was reported by DAVID for each significantly enriched pathway.

## Data Availability

The datasets presented in this study can be found in online repositories. The names of the repository/repositories and accession number(s) can be found in the article/Supplementary Material.
