# Supplementary material for: Gene representation in scRNA-seq is correlated with common motifs at the 3′ end of transcripts
Source: Front Bioinform. 2023 May 15;3:1120290. doi: 10.3389/fbinf.2023.1120290 (PMC10226423; doi:10.3389/fbinf.2023.1120290)
Supplement: Supplementary file 1 [file DataSheet1.PDF]

Gene representation in scRNA-seq is correlated with common motifs at the 3' end of transcripts

Xinling Li[1], Greg Gibson[2], Peng Qiu[1]

Supplementary Figure 1: page 2
Supplementary Figure 2: page 3
Supplementary Figure 3: page 4
Supplementary Figure 4: page 5
Supplementary Figure 5: page 6
Supplementary Table 1: page 7-8

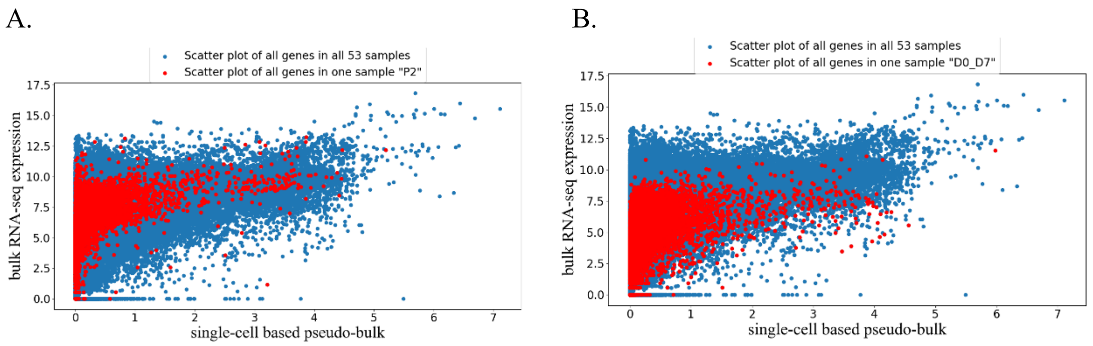

Supplementary Figure 1. Scatter plot visualization of paired bulk and pseudo-bulk data for 53 paired samples. The data preprocessing procedure of both bulk RNA-seq and scRNA-seq are the same as the ones in the main text, except that the bulk RNA-seq was not quantile normalized. (A) Sample “P2” is highlighted in red. (B) Sample “D0_D7” is highlighted in red.

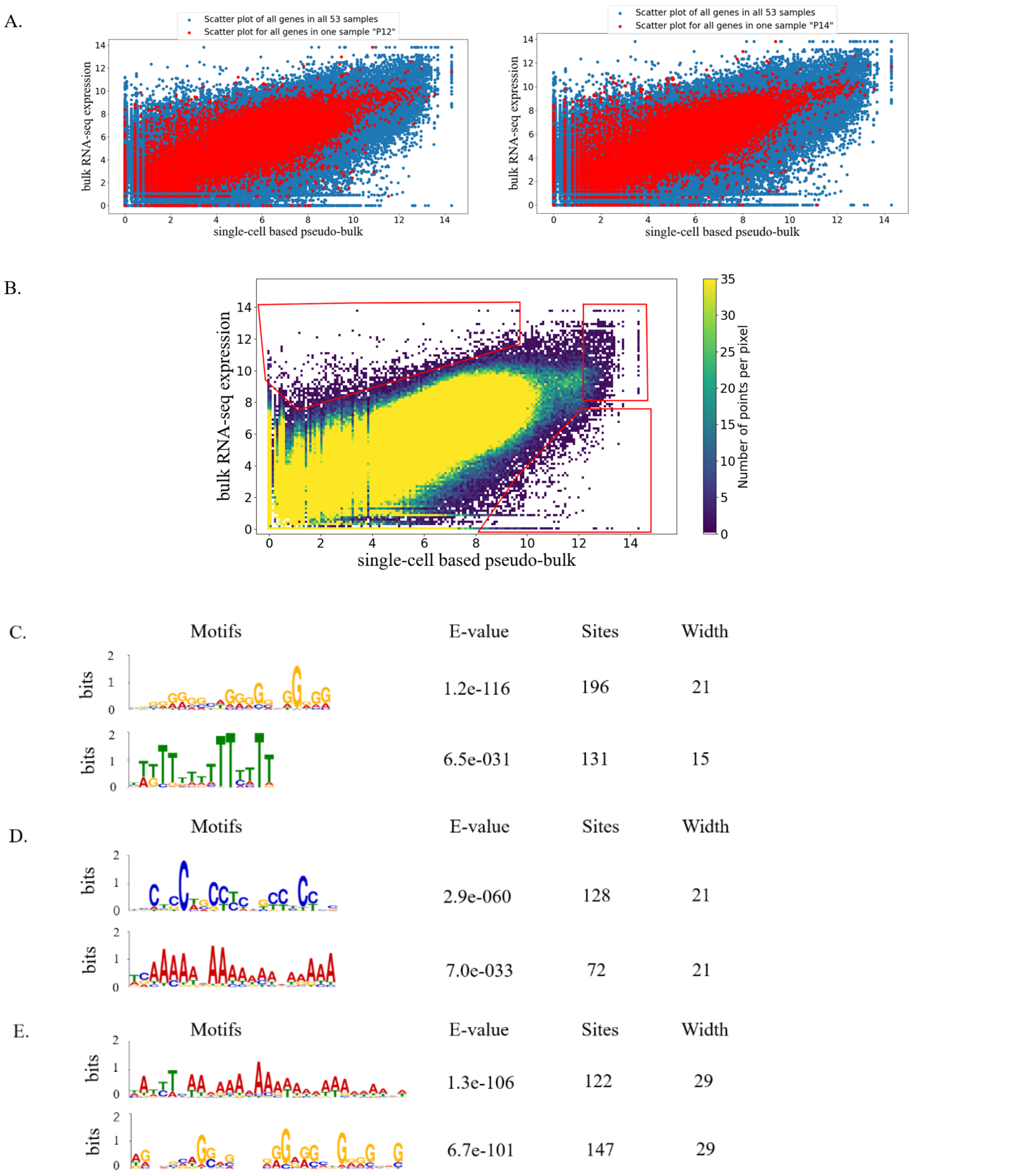

Supplementary Figure 2. Scatter plot visualization of paired bulk and pseudo-bulk data for 53 paired samples (A) using the same data preprocessing procedure of bulk RNA-seq data in the main text. For each scRNA-seq sample, sum of counts across all cells for each gene was first calculated. Then the total count of each gene was divided by sum of total counts of all genes in the sample and multiplied by 50,000,000. Next, median-of-ratios normalization was performed on processed scRNA-seq samples from each study using DESeq2. Then, natural log transformation was performed followed by quantile normalization. Density plot of scatterplot of 53 paired samples with gates indicating candidate genes in three aspects (B) together with significantly enriched motifs of genes occur more than once in upper-left (C), upper-right (D), and bottom (E) gates. The total number of dots in the three gates are 1,174, 1,603, and 886, respectively. The number of genes that occurred more than once in the three gates are 200, 118, and 148, respectively.

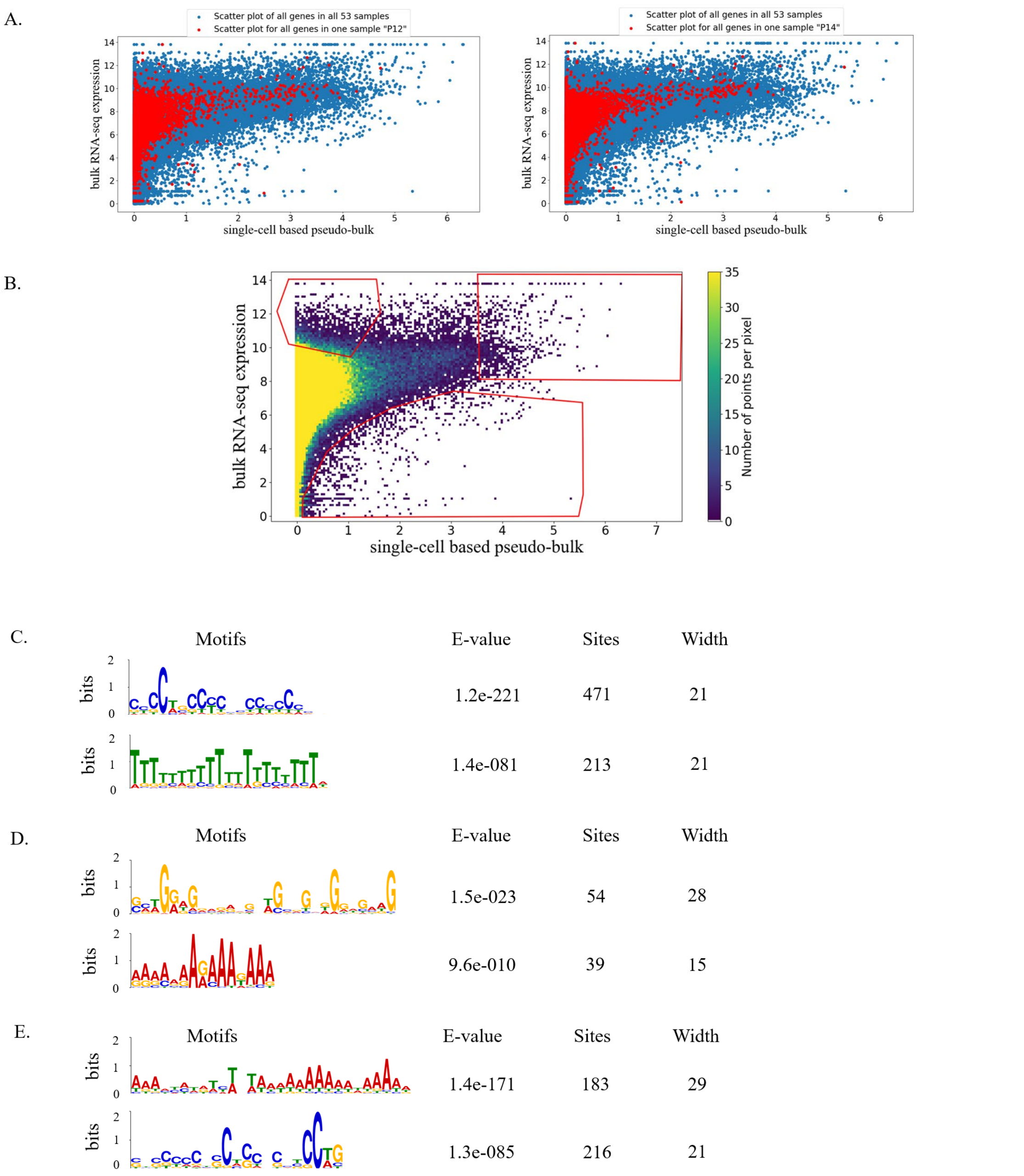

Supplementary Figure 3. Scatter plot visualization of paired bulk and pseudo-bulk data for 53 paired samples (A) using the same data preprocessing procedure of bulk RNA-seq data in the main text. For scRNA-seq, normalization was performed by SCTransform instead of Seurat and all other steps are the same as the main text. Density plot of scatterplot of 53 paired samples with gates indicating candidate genes in three aspects (B) together with significantly enriched motifs of genes occur more than once in upper-left (C), upper-right (D), and bottom (E) gates. The total number of dots in the three gates are 3,880, 734, and 1,043, respectively. The number of genes that occurred more than once in the three gates are 555, 102, and 197, respectively.

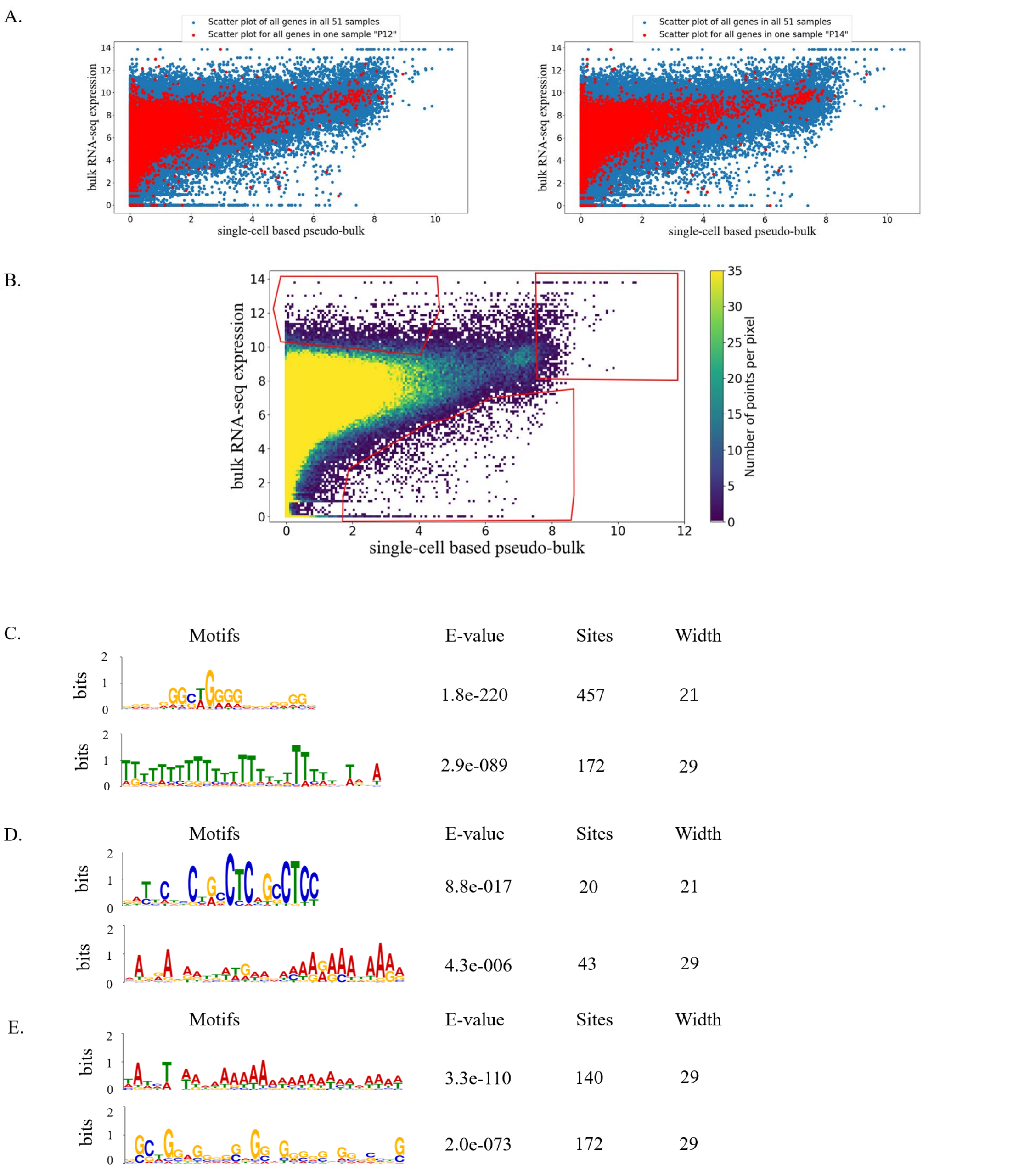

Supplementary Figure 4. Scatter plot visualization of paired bulk and pseudo-bulk data for 51 paired samples (A) using the same data preprocessing procedure of bulk RNA-seq data in the main text. For scRNA-seq, normalization was performed by Linnorm instead of Seurat and all other steps are the same as the main text. Density plot of scatterplot of 51 paired samples with gates indicating candidate genes in three aspects (B) together with significantly enriched motifs of genes occur more than once in upper-left (C), upper-right (D), and bottom (E) gates. The total number of dots in the three gates are 3,184, 816, and 963, respectively. The number of genes that occurred more than once in the three gates are 435, 63, and 163, respectively.

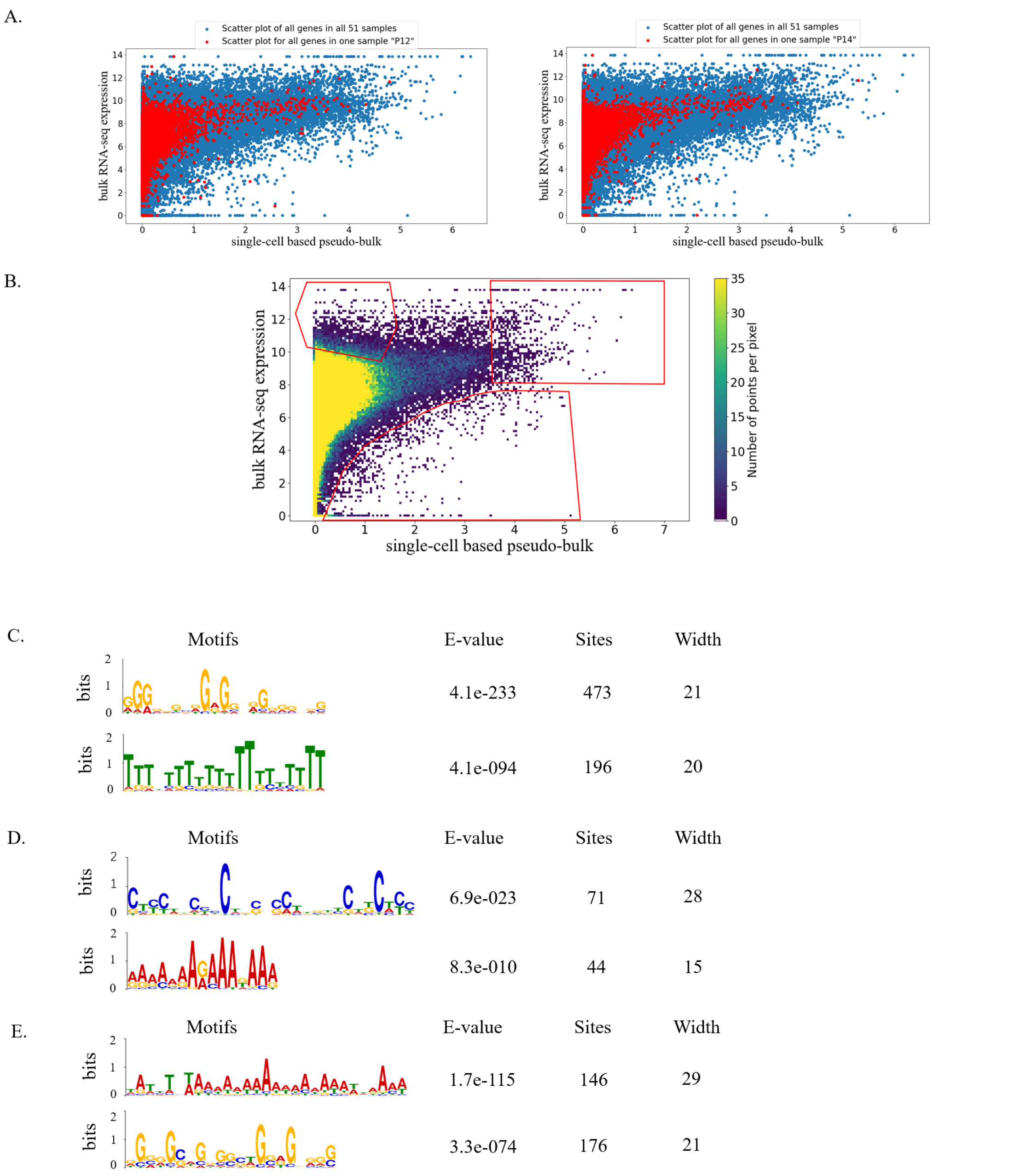

Supplementary Figure 5. Scatter plot visualization of paired bulk and pseudo-bulk data for 51 paired samples (A) using the same data preprocessing procedure of bulk RNA-seq data in the main text. For scRNA-seq, normalization was performed by scran instead of Seurat and all other steps are the same as the main text. Density plot of scatterplot of 51 paired samples with gates indicating candidate genes in three aspects (B) together with significantly enriched motifs of genes occur more than once in upper-left (C), upper-right (D), and bottom (E) gates. The total number of dots in the three gates are 3,568, 755, and 826, respectively. The number of genes that occurred more than once in the three gates are 482, 99, and 155, respectively.

**Bulk RNA-seq samples**	**scRNA-seq samples**
GSM4568149 N1_RNA-seq	GSM4568340 N1_scRNA-seq
GSM4568150 N3_RNA-seq	GSM4568342 N3_scRNA-seq
GSM4568151 P2_RNA-seq	GSM4568346 P2_scRNA-seq
GSM4568152 P3_RNA-seq	GSM4568347 P3_scRNA-seq
GSM4568153 P4_RNA-seq	GSM4568348 P4_scRNA-seq
GSM4568154 P5_RNA-seq	GSM4568349 P5_scRNA-seq
GSM4568155 P6_RNA-seq	GSM4568350 P6_scRNA-seq
GSM4568156 P7_RNA-seq	GSM4568351 P7_scRNA-seq
GSM4568157 P8_RNA-seq	GSM4568352 P8_scRNA-seq
GSM4568158 P9_RNA-seq	GSM4568353 P9_scRNA-seq
GSM4568159 P10_RNA-seq	GSM4568354 P10_scRNA-seq
GSM4568160 P11_RNA-seq	GSM4568355 P11_scRNA-seq
GSM4568161 P12_RNA-seq	GSM4568356 P12_scRNA-seq
GSM4568162 P13_RNA-seq	GSM4568357 P13_scRNA-seq
GSM4568163 P14_RNA-seq	GSM4568358 P14_scRNA-seq
GSM4916467 Basal population<br>GSM4916468 LP population<br>GSM4916469 ML population<br>(all from patient 0233)	GSM4909265<br>Normal Total cells from Patient 0233
GSM4916475 Basal population<br>GSM4916476 LP population<br>GSM4916477 ML population<br>(all from patient 1105)	GSM4909260<br>Normal Epithelial cells from Patient 1105
GSM4916494 Basal population<br>GSM4916493 LP population<br>GSM4916496 ML population<br>(all from patient 0408)	GSM4909259<br>Normal Epithelial cells from Patient 0408
CID3586	GSM5354513 CID3586
CID3838	GSM5354514 CID3838
CID3921	GSM5354515 CID3921
CID3941	GSM5354516 CID3941
CID3946	GSM5354517 CID3946
CID3948	GSM5354518 CID3948
CID3963	GSM5354519 CID3963
CID4040	GSM5354520 CID4040
CID4066	GSM5354521 CID4066b
CID4067	GSM5354522 CID4067
CID4290A	GSM5354523 CID4290A
CID4398	GSM5354524 CID4398
CID44041	GSM5354525 CID44041
CID4461	GSM5354526 CID4461
CID4463	GSM5354527 CID4463

CID4465	GSM5354528 CID4465
CID4471	GSM5354529 CID4471
CID4495	GSM5354530 CID4495
CID4513	GSM5354533 CID4513
CID4515	GSM5354534 CID4515
CID4523	GSM5354536 CID4523
CID4530N	GSM5354537 CID4530N
CID4535	GSM5354538 CID4535
CID44971	GSM5354531 CID44971
GSM4509079 RNA-seq_Fibroblast-D7_rep1	GSM4546545 SC_RNA-seq_D0_D7
GSM4509092 RNA-seq_t2iLGoY-D13_rep1	GSM4546546 SC_RNA-seq_Naive
GSM4509107 RNA-seq_Primed-D13_rep1	GSM4546547 SC_RNA-seq_Primed
GSM4509095 RNA-seq_RSeT-D13_rep1	GSM4546548 SC_RNA-seq_RSet
GSM2896739 451Lu_bulk_parental_rep1	GSM2897333 451Lu_10x_singlecell_parental
GSM2896741 451Lu_bulk_resistant_rep1	GSM2897334 451Lu_10x_singlecell_resistant
GSM4041653 3 Cell-Line-mixture-Bulk	GSM4041646 3 Cell-line mixture
GSM4272911 cc011-16_CD66N	GSM4272915 cc04
GSM4272903 cc02-17_CD66N	GSM4272916 cc05
GSM3703348<br>Mixture 1: 100% HEK, 0% Jurkat	https://www.10xgenomics.com/resources/datasets/293-t-cells-1-standard-1-1-0
GSM3703361<br>Mixture 14: 0% HEK, 100% Jurkat	https://www.10xgenomics.com/resources/datasets/jurkat-cells-1-standard-1-1-0

Supplementary Table 1. Accession number of individual sample pairs for all datasets in this study. Each bulk RNA-seq and scRNA-seq pair was either from the same tissue of the same patient, or the same tissue source, or the same cell lines.
